# TRPM3 channel stimulated by pregnenolone sulphate in synovial fibroblasts and negatively coupled to hyaluronan

**DOI:** 10.1186/1471-2474-11-111

**Published:** 2010-06-04

**Authors:** Coziana Ciurtin, Yasser Majeed, Jacqueline Naylor, Piruthivi Sukumar, Anne A English, Paul Emery, David J Beech

**Affiliations:** 1 Institute of Membrane & Systems Biology, Faculty of Biological Sciences, University of Leeds, Leeds, LS2 9JT, UK; 2 Carol Davila University of Medicine & Pharmacy, Bucharest, Romania; 3 Academic Section of Musculoskeletal Disease, University of Leeds, Chapel Allerton Hospital, Leeds LS7 4SA, UK

## Abstract

**Background:**

Calcium-permeable channels are known to have roles in many mammalian cell types but the expression and contribution of such ion channels in synovial cells is mostly unknown. The objective of this study was to investigate the potential relevance of Transient Receptor Potential Melastatin 3 (TRPM3) channel to fibroblast-like synoviocytes (FLSs) of patients with rheumatoid arthritis.

**Methods:**

The study used RT-PCR and immunofluorescence to detect mRNA and protein. Intracellular calcium measurement detected channel activity in a FLS cell-line and primary cultures of FLSs from patients with rheumatoid arthritis. Enzyme-linked immunosorbent assays measured hyaluronan.

**Results:**

Endogenous expression of TRPM3 was detected. Previously reported stimulators of TRPM3 sphingosine and pregnenolone sulphate evoked sustained elevation of intracellular calcium in FLSs. The FLS cell-line showed an initial transient response to sphingosine which may be explained by TRPV4 channels but was not observed in FLSs from patients. Blocking antibody targeted to TRPM3 inhibited sustained sphingosine and pregnenolone sulphate responses. Secretion of hyaluronan, which contributes adversely in rheumatoid arthritis, was suppressed by pregnenolone sulphate in FLSs from patients and the effect was blocked by anti-TRPM3 antibody.

**Conclusions:**

The data suggest that FLSs of patients with rheumatoid arthritis express TRPM3-containing ion channels that couple negatively to hyaluronan secretion and can be stimulated by pharmacological concentrations of pregnenolone sulphate.

## Background

Transient Receptor Potential (TRP) was discovered in *Drosophila melanogaster *as a component of the visual response to bright light. Subsequently, 28 related genes were discovered in mammals, found to be widely expressed and shown to be involved in various chemical and temperature sensing mechanisms [1,2]. The mammalian TRP channels are divided into subgroups according to amino acid sequence similarities; one subgroup is the M (melastatin) subgroup of which there are eight members, while other subgroups include the V (vanilloid) and C (canonical) subgroups. We previously reported on expression and function of TRPC channels in fibroblast-like synoviocytes (FLSs) [3]. There have also been indications of TRPV1, TRPV4 and TRPM8 functions in FLSs [4,5], but regulation and importance of TRP channels in the synovial lining is relatively unknown. Here we focus on TRPM3 [6-11]. When over-expressed in cell-lines TRPM3 forms Ca^2+^-permeable non-selective cationic channels at the plasma membrane that can be stimulated by factors including sphingosine and pregnenolone [8,10]. Activity of endogenous TRPM3 channels and the biological functions of such channels are poorly understood but a recent seminal study showed expression in pancreatic β-cells and suggested coupling of pregnenolone to insulin secretion via TRPM3 [10]. Pregnenolone is derived from cholesterol and the precursor for steroid hormones including the corticosteroids, androgens, oestrogen and progesterone. It is sulphated *in vivo *and the sulphated form (pregnenolone sulphate) is suggested to have biological actions, including through glutamate and γ-aminobutyric acid receptors of neurones [12,13].

Rheumatoid arthritis (RA) is a common and debilitating disease affecting large numbers of people world-wide. Inflammation of the synovial lining is a striking feature of the disease and is contributed to strongly by hyperplasia of FLSs [14-16]. Hyaluronan is secreted by FLSs and is a well-recognised lubricant of physiological joints, but excess hyaluronan from FLSs and degradation products of hyaluronan contribute to RA [17]. Furthermore, hyaluronan from other cell types may have general adverse consequences in inflammatory conditions, including in atherosclerosis [18,19].

In this study we investigated TRPM3 and its stimulators by using a model FLS cell-line and primary cultures of FLSs from patients diagnosed with RA.

## Methods

### HIG-82 cells

HIG-82 cells are a fibroblast-like synoviocyte (FLS) cell-line from soft tissue lining the knee joints of rabbits [20,3]. Cells were cultured in Ham's F-12 medium (Gibco, UK) containing 10% fetal calf serum, 100 units·ml^-1 ^penicillin and 100 μg·ml^-1 ^streptomycin. Cells were maintained in a humidified atmosphere of 5% CO2 in air and re-plated on 96-well plates 24 hr prior to experiments. The cells stained positively for CD55 and negatively for CD68, supporting their identification as FLS-like cells [3].

### Synovial tissue

Tissue biopsies were obtained during arthroscopy from the synovial lining of knee joints of patients diagnosed with RA at the Academic Unit of Musculoskeletal Disease, Chapel Allerton Hospital, Leeds. Ethical approval was given by the Local Ethics Committee. Patient informed consent was obtained. No attempt was made to correlate findings with the severity of the RA or other patient characteristics. Patient therapy was standard disease-modifying anti-rheumatic drugs but not anti-tumor necrosis factor (TNF) therapies or high-dose steroids.

### Primary cultures of FLSs

Synovial biopsies were washed with phosphate-buffered saline and digested with 0.25% type 1A collagenase (Stem Cell Technologies, UK) for 4 hr at 37°C and FLSs were then cultured in DMEM/F-12 + Glutamax (Gibco, UK) containing 10% fetal calf serum, 100 units·ml^-1 ^penicillin and 100 μg·ml^-1 ^streptomycin. FLSs were maintained in a humidified atmosphere of 5% CO_2 _in air and replated on multi-well plates prior to experiments.

### Over-expression of TRPV4

Chinese hamster ovary (CHO) K1 cells stably expressing human TRPV4 were maintained in Ham's F12 (Gibco, UK) in the presence of 1 mg ml^-1 ^G418 (Sigma, UK).

### RT-PCR

Total RNA was extracted using a Tri-reagent protocol followed by DNase I (Ambion) treatment. 1 μg of total RNA was used for reverse transcription (RT) based on oligo-dT primers and AMV RT enzyme. TRPM3 PCR primers were (5'-3') AGCAGTTCTACCTAACGT (forward primer) and CTTGCTCGACTAGACTTG (reverse primer). The expected product size was 268 bp. The specificity of the RT-PCR was verified by reactions without RT (-RT) and by sequencing of the PCR product (University of Leeds, UK).

### Labeling with antibody

Tissue sections (4 μm thick) were cut from snap-frozen biopsy samples, fixed with acetone and stored at -80°C until use (immunofluorescence labeling) or fixed with 1% paraformaldehyde (immunohistochemistry). Staining was according to standard protocols: Briefly, sections were incubated with primary antibody over night at 4°C and secondary antibody (goat anti-rabbit IgG-FITC or HRP) for 1 hr at room temperature. Slides were mounted (Vector Labs, UK) and imaged with a confocal or bright-field microscope. Anti TRPM3 (TM3E3) and anti-CD55 (clone 67, Serotec) primary antibodies were used at 1:500 and 1:200 dilutions respectively. TM3E3 was described previously [11].

### Hyaluronan secretion

Primary FLSs were cultured in 6 well plates for 24 hr without serum starvation and then fresh medium was added containing PregS, dexamethasone, TNF or vehicle. Incubations with antibodies occurred in culture medium for 2 hr prior to addition of compounds. Supernatants were collected after 24 hr, frozen and analysed using an enzyme-linked immunosorbent assay (ELISA) that detects hyaluronan (HA ELISA kit, Corgenix).

### Intracellular Ca^2+ ^measurement

Ca^2+ ^was measured using a FlexStation II controlled by SoftMax Pro 4.7.1 software (Molecular Devices, USA). Cells were plated at 60-70% confluence on 96-well square-bottomed clear plates (Nunclon, UK) 24 hr before experiments. Immediately prior to recording, cells were incubated for 1 hr at 37°C in SBS (see below) containing 10 μM fura-2 acetoxymethyl ester and then washed with SBS 2-3 times before adding fresh SBS. Incubations with TM3E3 antibody occurred for 2 hr prior to the incubation with the fluorescent dye, during the incubation and the washing period (i.e. for 3.5 hr prior to recording data). Excess TM3E3 was removed immediately prior to experiments. Excitation light alternated between 340 and 380 nm, while emission was filtered at 510 nm. Experiments were at room temperature (21 ± 3°C). Standard bath solution (SBS) contained (mM): 130 NaCl, 5 KCl, 1.2 MgCl_2_, 1.5 CaCl_2_, 8 glucose and 10 Hepes (titrated to pH 7.4 using NaOH and adjusted to 290 mOsm using mannitol). Ca^2+^-free SBS was SBS in which CaCl_2 _was replaced by 0.4 mM EGTA.

### Reagents

All reagents were from Sigma (UK) or BDH (British Drug House, Poole, UK). Sphingosine was prepared as a 20 mM stock in 100% ethanol and stored at -20°C, pregnenolone sulphate as a 100 mM stock in DMSO and stored at 4°C. ATP was prepared as a 100 mM stock in H_2_O and lysophosphatidylcholine (LPC) was a 100 mM stock in 100% methanol; both were stored at -20°C.

### Data analysis

Data are expressed as mean ± s.e.m. and paired data sets compared using two-tailed *t*-tests. For FlexStation recordings, the numbers of experiments are indicated as N/n, where N is the number of wells used in the 96-well plate and n is the number of independent experiments (i.e. different 96-well plates and batches of cells). All data from primary cultures of FLSs derived from at least 3 independent patient samples. Statistical significance is indicated by probability (P-value) of *P < 0.05 unless specified; NS indicates no significant differences. Data were handled, analysed and prepared for presentation using Origin software (Origin Lab Corporation, Northampton, CA, USA).

## Results

### Endogenous expression of TRPM3

**Figure 1:**
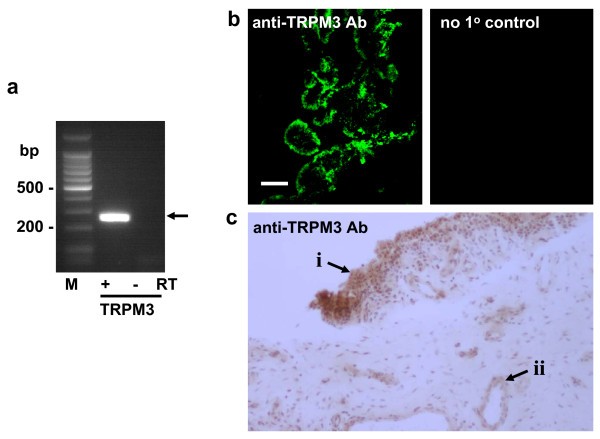
**Expression of TRPM3 in FLSs of synovium in rheumatoid arthritis**. (**a**) PCR products from analysis of mRNA isolated from primary cultured FLSs from patients with (+) and without (-) prior reaction with reverse transcriptase (RT). On the left is the DNA marker ladder (M). The expected TRPM3 mRNA product size is indicated with an arrow. (**b**) Serial sections of RA synovial biopsy labeled with the primary (1°) anti-TRPM3 antiserum present (left image) or absent (right image). Positive labeling is green. The scale bar is 50 μm. (**c**) A section of RA synovial biopsy labeled with anti-TRPM3 antiserum (brown) and counter stained with haematoxylin to label cell nuclei (purple). The lining of the synovium (i) and a blood vessel (ii) are indicated by arrows.

Messenger RNA encoding TRPM3 was detected in FLSs from patients with RA (Fig 1a). The TRPM3 protein was expressed endogenously because anti-TRPM3 antibody [11] labeled cells at the edge of the synovial lining in synovial biopsies (Fig 1b); the cells were CD55 positive (data not shown), supporting identification as FLSs [3]. TRPM3 could also be detected in the sub-lining cell layer and in blood vessels of the joint (Fig 1c).

### Ca^2+^-entry evoked by sphingosine in HIG-82 cells

Because of limited supply of synovial biopsies we first sought to investigate the functional relevance of TRPM3 expression through experiments on a FLS cell-line (HIG-82 cells), a proposed model system of proliferating FLSs in joints. As with synovial biopsies, there was labeling of the cells by anti-TRPM3 (data not shown) and anti-CD55 antibodies [3].

**Figure 2:**
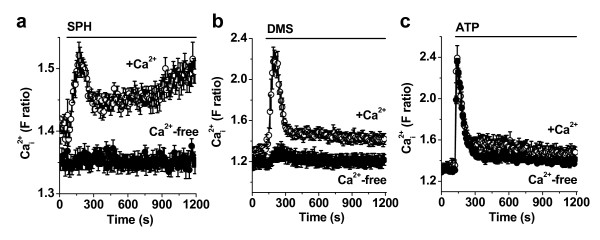
**Sphingosine-evoked Ca^2+ ^signals in HIG-82 cells**. Data were generated by intracellular Ca^2+ ^measurement. Responses elicited by (**a**) sphingosine (SPH, 20 μM), (**b**) dimethylsphingosine (DMS, 20 μM) or (**c**) adenosine triphosphate (ATP, 100 μM) were tested in either 1.5 mM Ca^2+^-containing (+Ca^2+^) or Ca^2+^-free extracellular solution. Responses are each representative of 3 (n) independent experiments.

TRPM3 channels are suggested to be Ca^2+ ^permeable and so we studied channel activity using an intracellular Ca^2+ ^indicator. Cells were exposed to sphingosine because it has been suggested to stimulate human TRPM3 over-expressed in HEK 293 cells [8]. In HIG-82 cells, sphingosine elicited a rise in the intracellular Ca^2+ ^concentration in two phases - an initial transient phase followed by a sustained phase (Fig 2a). Dimethylsphingosine also stimulated over-expressed human TRPM3 [8] and elicited transient followed by sustained Ca^2+ ^responses in HIG-82 cells (Fig 2b).

The transient and sustained phases of the sphingosine and dimethylsphingosine responses depended on the presence of extracellular Ca^2+ ^(Fig 2a, b), which suggested that both phases were due to Ca^2+^-influx. It was conceivable however that removal of extracellular Ca^2+ ^caused depletion of intracellular Ca^2+ ^stores, leading to the false impression of Ca^2+^-influx. To investigate this possibility we applied extracellular ATP to activate G protein-coupled purinoceptors. ATP elicited a predominantly transient rise in Ca^2+ ^that was unaffected by removal of extracellular Ca^2+ ^(Fig 2c), showing that ATP acted by evoking Ca^2+^-release and that Ca^2+^-stores had not been inadvertently depleted. The data support the conclusion that sphingosine evoked Ca^2+ ^influx.

### Role of TRPM3 in the sustained sphingosine response of HIG-82 cells

**Figure 3:**
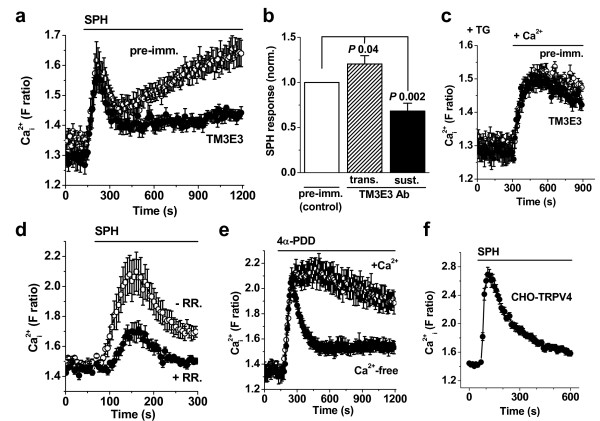
**Inhibition of sustained sphingosine responses by anti-TRPM3 antibody in HIG-82 cells**. Data were generated by intracellular Ca^2+ ^measurement. **(a) **Cells were pre-treated with 1:4000 dilution of pre-immune serum (pre-imm.) or anti-TRPM3 antiserum before observing responses to 20 μM sphingosine (SPH). **(b) **Summary normalised data from 8 independent experiments showing inhibitory effect of TM3E3 on the sustained response to SPH. **(c) **Lack of effect of TM3E3 on Ca^2+ ^entry in cells store-depleted by 1 μM thapsigargin (TG) for 30 min in Ca^2+^-free extracellular solution followed by re-addition of 1.5 mM Ca (+Ca^2+^); representative of N/n = 24/3. (**d**-**f**) Investigation of the transient response to SPH. **(d) **Effect of 10 μM ruthenium red (+RR.) on the SPH response in HIG-82 cells; representative of N/n = 12/3. **(e) **Responses to 10 μM 4α-phorbol 12,13-didecanoate (4α-PDD) in HIG-82 cells in the presence (+Ca^2+^) and absence of extracellular Ca^2+^; representative of N/n = 16/2. (**f**) Representative of 3 independent experiments, responses to sphingosine (SPH, 20 μM) in CHO cells stably expressing TRPV4.

It was previously described that the anti-TRPM3 antibody TM3E3 has an inhibitory effect on human TRPM3 over-expressed in HEK 293 cells [11]. We, therefore, investigated the effect of TM3E3 on the sphingosine response in HIG-82 cells. The sustained response to sphingosine was clearly suppressed (Fig 3a, b). The transient response to sphingosine was either unaffected (Fig 3a) or slightly potentiated, with the mean data just reaching statistical significance (Fig 3b). Evidence for specificity of TM3E3 came from its lack of effect on the Ca^2+ ^response evoked by the Ca^2+^-ATPase inhibitor thapsigargin (Fig 3c, and see below). The data suggest that a substantial component of the sustained response to sphingosine was due to stimulation of TRPM3-containing Ca^2+^-influx channels.

### Potential role of TRPV channels in the transient sphingosine response of HIG-82 cells

To gain insight into the TRPM3-independent transient response we investigated its sensitivity to ruthenium red, which is a non-specific inhibitor of TRP channels [1]. Ruthenium red was inhibitory (Fig 3d), consistent with the transient response being substantially due to Ca^2+^-influx and suggesting dependence on TRP channels [1]. Ruthenium red is best known as an inhibitor of the TRPV subfamily of TRP channels. Therefore, we applied TRPV channel stimulators in an effort to mimic the transient effect of sphingosine.

4α-Phorbol 12,13-didecanoate (4-α-PDD) is an agonist at TRPV4 channels and appears to be specific for this channel type [21]. The compound elicited a Ca^2+ ^response (Fig 3e), which suggested that functional TRPV4 channels were expressed in the HIG-82 cells. Removal of extracellular Ca^2+ ^reduced the sustained response to 4-α-PDD, although a transient response remained (Fig 3e). The data suggest that TRPV4 channels are functional at the plasma membrane. Consistent with the notion that sphingosine caused transient Ca^2+ ^entry in HIG-82 cells because it stimulated TRPV4, sphingosine evoked a similar transient rise of the intracellular Ca^2+ ^concentration in a cell-line stably expressing the clone of human TRPV4 (Fig 3f).

### Effect of sphingosine on primary FLSs

**Figure 4:**
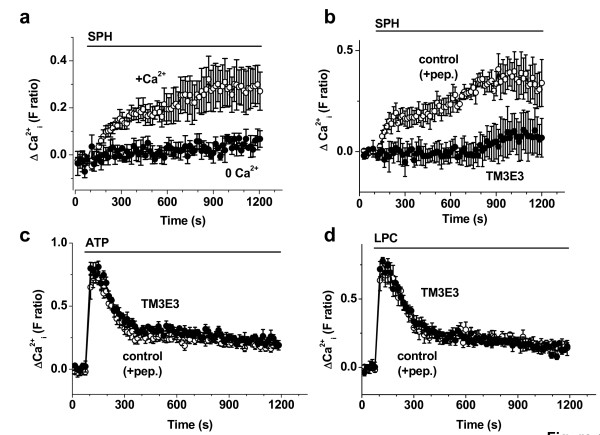
**Ca^2+^-entry evoked by sphingosine (SPH) in primary cultures of FLSs from patients with RA**. (**a**) Intracellular Ca^2+ ^measurement showing the effect of external application of 20 μM SPH in the presence and absence of extracellular Ca^2+^. (**b**-**d**) Intracellular Ca^2+ ^measurements showing responses to externally applied substances: (**b**) 20 μM SPH, (**c**) 100 μM adenosine triphosphate (ATP), and (**d**) 30 μM lysophosphatidylcholine (LPC). In paired experiments on the same 96-well plate, responses were compared after pre-treatment with anti-TRPM3 antibody (TM3E3) or TM3E3 preadsorbed to its antigenic peptide ("+pep."; control group). (**a**-**d**) All data points are N = 8 and representative of 3 (n) independent experiments.

Sphingosine responses were also investigated in primary cultures of FLSs from patients with RA to determine the relevance of the HIG-82 cell data. As with HIG-82 cells, sphingosine elicited sustained Ca^2+ ^responses that depended on extracellular Ca^2+ ^(Fig 4a). In contrast to HIG-82 cells, there was no transient response to sphingosine (Fig 4a). Consistent with dominant contribution of sustained Ca^2+ ^entry and TRPM3 channels, TM3E3 antibody inhibited the response (Fig 4b). TM3E3 did not act non-specifically because it had no effect on Ca^2+ ^responses to ATP or lysophosphatidylcholine (LPC) (Fig 4c, d).

### Effect of pregnenolone sulphate on primary FLSs

**Figure 5:**
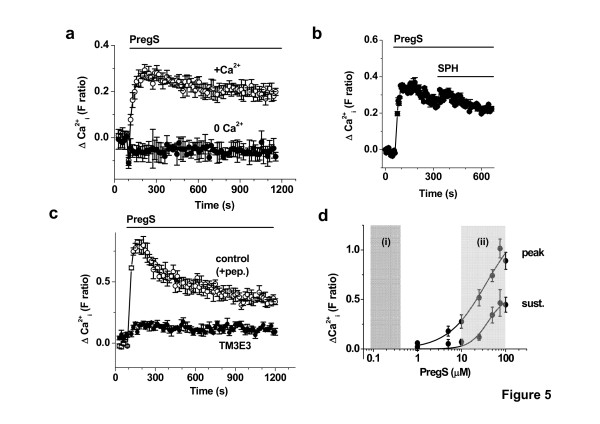
**Ca^2+^-entry evoked by pregnenolone sulphate (PregS) in primary cultures of FLSs from patients with RA**. (**a**) Intracellular Ca^2+ ^measurement showing the effect of external application of 100 μM PregS in the presence and absence of extracellular Ca^2+^. (**b**) As for (**a**) but the effect of PregS (100 μM) and then addition of 20 μM SPH. (**c**) As for (**a**) but paired experiments on the same 96-well plate where responses were compared after pre-treatment with anti-TRPM3 antibody (TM3E3) or TM3E3 preadsorbed to its antigenic peptide ("+pep."; control group). (**a**-**c**) All data points are N = 8 and representative of 3 (n) independent experiments. (**d**) Concentration-dependence of Ca^2+^-entry evoked by PregS. Mean data are shown for measurement of the peak and sustained responses to PregS (e.g. see (**c**)); 4 (n) independent experiments. The fitted curves are Hill equations with mid-points at 35.7 μM (peak) and 39.5 μM (sustained). The shaded area on the left (i) indicates the highest concentrations reported for endogenous PregS [22,23], while the shaded area on the right (ii) indicates the higher concentrations of plasma PregS predicted on oral administration of 100 mg daily to adult humans.

A recently-reported stimulator of TRPM3 channels is pregnenolone sulphate [10]. Pregnenolone sulphate elicited a robust rise in Ca^2+ ^in FLSs from patients with RA (Fig 5a). The response was abolished in the absence of extracellular Ca^2+ ^(Fig 5a) and the response to sphingosine was not additive on top of the pregnenolone sulphate response (Fig 5b), suggesting that pregnenolone sulphate and sphingosine stimulate the same Ca^2+ ^influx pathway. In support of this hypothesis, TM3E3 antibody strongly inhibited the pregnenolone sulphate response (Fig 5c). Concentration-response curves were constructed to determine if the effect of pregnenolone sulphate on Ca^2+^-influx might be relevant to physiological concentrations of the steroid. Concentrations of >1 μM pregnenolone sulphate were effective at elevating the intracellular Ca^2+ ^concentration (Fig 5d), which are above the known physiological concentrations of this steroid [22,23].

### Functional roles of TRPM3 and pregnenolone sulphate responses in primary FLSs

**Figure 6:**
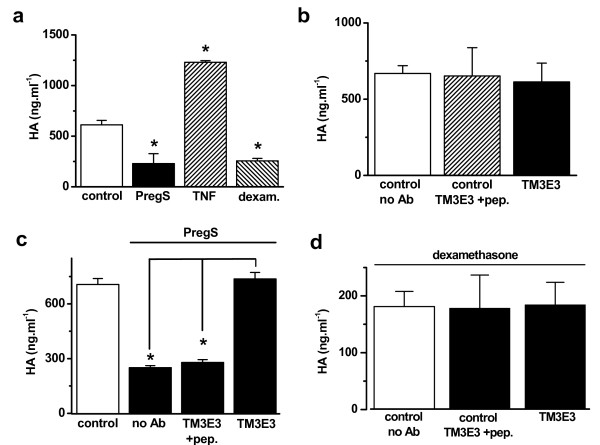
**Negative coupling to hyaluronan secretion in primary cultures of FLSs from patients with RA**. Data are from measurements of hyaluronan derived from cells of 6 (a-c) or 3 (d) patient samples. (a) Mean effects of 100 μM pregnenolone sulphate (PregS), 10 ng/ml tumor necrosis factor (TNF) and 1 μg/ml dexamethasone; 'control' was the solvent. (b) Mean data showing no effect of anti-TRPM3 antibody (TM3E3) on basal hyaluronan secretion; one control was without TM3E3 (no Ab) and another was from cells pretreated with TM3E3 preadsorbed to its antigenic peptide (TM3E3 + pep.). (c) As for (b) but showing the effect 100 μM PregS under the different antibody (Ab) conditions; the control was cells without PregS. (d) Failure of TM3E3 to protect against the inhibitory effect of 1 μg/ml dexamethasone.

Hyaluronan is secreted by FLSs and its secretion is excessive and pro-inflammatory in RA [17]. We hypothesized that Ca^2+ ^might modulate the secretion. Hyaluronan secretion was, therefore, measured and first investigated using the established positive controls tumour necrosis factor and dexamethasone that stimulate and inhibit hyaluronan secretion respectively; both compounds acted as expected (Fig 6a). Like dexamethasone, pregnenolone sulphate inhibited hyaluronan secretion (Fig 6a). TM3E3 alone had no effect (Fig 6b) but prevented the action of pregnenolone sulphate (Fig 6c). TM3E3 was ineffective when it had been preadsorbed to its antigenic peptide (Fig 6c), showing that its effect depended on the TRPM3 peptide recognition site of the antibody. Although dexamethasone also inhibited hyaluronan secretion (Fig 6a), its effect was unchanged by TM3E3 (Fig 6d). The data suggest that stimulation of TRPM3 inhibited hyaluronan production or secretion.

## Discussion

The data add to an emerging picture of multiple types of TRP channel in fibroblast-like synoviocytes (FLSs) [3,4], specifically suggesting that TRPM3 channels enable sustained Ca^2+ ^responses to sphingosine and pregnenolone sulphate. Of potential functional relevance was the observation that pregnenolone sulphate acted via TRPM3 to negatively modulate hyaluronan secretion, independently of the pathway activated by glucocorticoids. The data, therefore, suggest a molecular basis for therapeutic benefit of pregnenolone in rheumatoid arthritis, as initially suggested in publications of the 1950s and commonly suggested in the contemporary field of dietary supplementation.

We cannot exclude that endogenous concentrations of pregnenolone sulphate (or a related steroid) become sufficiently high in RA to stimulate TRPM3 in FLSs but there is currently no evidence to support such a hypothesis. Small effects of the related steroids pregnenolone, dehydroepiandrosterone (DHEA) and DHEA sulphate (DHEA-S) were observed, but again the concentrations are unlikely to be physiologically relevant. Physiological DHEA-S concentrations are high (3-4 μM in patients with RA [24]) but we observed only minor effects of 100 μM DHEA-S on Ca^2+ ^in primary cultures of FLSs and no effects of lower concentrations (C Ciurtin *et al*, unpublished data).

There is greater reason to suggest that the effect of pregnenolone sulphate on Ca^2+ ^entry in FLSs is pharmacologically interesting because pregnenolone (much of which is sulphated *in vivo*) is self-administered as a dietary supplement and informally considered to have general health-giving benefits, including in RA. There are on-going clinical trials of pregnenolone or related steroids to rigorously test the benefits and adverse effects, but not yet in RA. Reasons why pregnenolone might be considered are as follows: (i) a range of small-scale studies published in the early 1950s suggest that pregnenolone may be of benefit in RA (Additional file 1); (ii) our data suggest a mechanistic basis for a beneficial action of pregnenolone in RA (i.e. inhibition of hyaluronan secretion via TRPM3 stimulation); (iii) Pregnenolone has been taken orally at high concentrations and remains readily available, yet major adverse effects are not apparent; (iv) Readily tolerated concentrations of pregnenolone are expected to generate sufficient plasma concentrations of pregnenolone sulphate to stimulate TRPM3 and inhibit hyaluronan secretion; (v) the mechanism of action of pregnenolone is distinct from that of dexamethasone and it is expected to lack the unwanted effects of glucocorticoids. Although pregnenolone is the precursor for all steroid hormones, administration of pregnenolone does not appear to elevate concentrations of sex steroids, presumably because of sulphation and rate-limiting steps in steroid hormone synthesis. A reason not to consider pregnenolone as potentially clinically relevant in RA is that the field has moved forward enormously since the studies of the 1950s. Can an endogenous steroid be seriously considered at time of major breakthroughs in biological (antibody) drug treatment? We suggest that it may have a valuable role to play but that rigorous clinical trials would be needed first. The potentially low cost of pregnenolone could be attractive to health providers and pregnenolone could confer a useful addition to therapeutic options during flare or during periods of remission after biological therapy.

It was reported that sphingosine and dimethylsphingosine stimulate over-expressed human TRPM3 [8] and we confirmed this observation when transiently expressing the same clone [11]. Another research group suggested that sphingosine is ineffective as a stimulator of TRPM3 and has non-specific effects [10]. We do not know the explanation for the difference but our data on endogenous TRPM3 of FLSs supports the view that sphingosine stimulates TRPM3; nevertheless, we have no strong argument for it as a physiological regulator of TRPM3.

We previously evaluated anti-TRPM3 antibody, TM3E3, against the function of human TRPM3 over-expressed in HEK 293 cells [11]. The antibody had a blocking effect in a variety of assays but it was striking that the effect was only partial (<50% inhibition). It was therefore surprising to find such a strong effect in FLS studies. The greater effect may have arisen because the FLS studies involved endogenous TRPM3, with normal expression and degradation mechanisms. In HEK 293 cell over-expression studies, expression is driven at a high rate, conferring rapid replacement of channels at the cell surface and possibly saturation of degradation mechanisms. We suspect that the antibody inhibits channels, at least in part, via a mechanism that requires channel internalization. Therefore, over-expression studies may under-estimate the degree of block because new channels replace antibody-bound channels and internalization of bound channels is slow.

## Conclusions

The data suggest expression and function of TRPM3 channels in cells that line the synovium of joints of patients suffering from rheumatoid arthritis. TRPM3 could also be detected in cells of the sub-lining layer and in small blood vessels of the joints, consistent with a recent report of vascular TRPM3 expression [25]. The channels may be stimulated by sphingosine or pregnenolone sulphate but the physiological relevance of these stimulators is uncertain. Instead we discuss arguments for further investigation of pregnenolone as a treatment for RA, acting in part through negative coupling to hyaluronan and potentially lacking unwanted effects of glucocorticoids. Overall, the data add to an emerging picture of TRP channel function in the synovium and suggest previously unrecognized regulation of hyaluronan secretion by ion channel activity at the plasma membrane.

## Abbreviations

TRP: Transient Receptor Potential; TRPM: Transient Receptor Potential Melastatin; FLS: fibroblast-like synoviocyte; RA: rheumatoid arthritis.

## Competing interests

The authors declare that they have no competing interests.

## Authors' contributions

CC and YM performed experiments and provided intellectual input, JN provided experimental tools and technical input, PS performed experiments, AE and PE provided and characterized tissue samples, and DJB provided the intellectual basis for the project, coordinated the project and wrote the paper. All authors read and approved the final manuscript.

## Pre-publication history

The pre-publication history for this paper can be accessed here:

http://www.biomedcentral.com/1471-2474/11/111/prepub

## Supplementary Material

Additional file 1 **Summary of reports on effects of pregnenolone administration on patients with rheumatoid arthritis**. Click here for file
