# Supplementary material for: TRPM3 channel stimulated by pregnenolone sulphate in synovial fibroblasts and negatively coupled to hyaluronan
Source: BMC Musculoskelet Disord. 2010 Jun 4;11:111. doi: 10.1186/1471-2474-11-111 (PMC2893450; doi:10.1186/1471-2474-11-111)
Supplement: Additional file 1 — Summary of reports on effects of pregnenolone administration on patients with rheumatoid arthritis. [file 1471-2474-11-111-S1.PDF]

ADDITIONAL INFORMATION

TRPM3 channel stimulated by pregnenolone sulphate in synovial fibroblasts and negatively coupled to hyaluronan

Coziana Ciurtin[†1,2], Yasser Majeed[†1], Jacqueline Naylor[1], Piruthivi Sukumar[1], Anne English[3], Paul Emery[3] & David J Beech[1]*

[1]Institute of Membrane & Systems Biology, Faculty of Biological Sciences, University of Leeds, Leeds, LS2 9JT, UK; [2]Carol Davila University of Medicine & Pharmacy, Bucharest, Romania; [3]Academic Section of Musculoskeletal Disease, University of Leeds, Chapel Allerton Hospital, Leeds LS7 4SA, UK.

[†] authors contributed equally

*author for correspondence

Professor David J Beech

Garstang Building

Faculty of Biological Sciences

Mount Preston Street

University of Leeds,

Leeds, LS2 9JT, England (UK)

Tel: +44-(0)-113-343-4323

Fax: +44-(0)-113-343-4228

Email: d.j.beech@leeds.ac.uk

Table I. Summary of reports on effects of pregnenolone administration on patients with rheumatoid arthritis.

Disease	Sample	Treatment	Outcome	Reference
rheumatoid arthritis	30 patients	300-700 mg daily pregnenolone or pregnenolone acetate tablets	24 improved (15 strong, 9 mild)	1
factory workers	>100 patients	45 mg pregnenolone in lactose vs placebo (lactose) tablets daily	Feeling of well-being, no adverse effect	2
rheumatoid arthritis	10 patients	400-500 mg daily or 200-300 mg i.m. daily<br>Mean total dose: 8650 mg pregnenolone	7 improved (6 mild, 1 strong)	3
rheumatoid arthritis	18 patients	400-500 mg p.o. daily and 100 mg i.m. daily for 2 weeks, followed by a maintenance dose of 400 mg p.o. daily and 100 mg pregnenolone i.m. twice a week, 4-10 weeks	7 improved (6 durable)<br>Better response in early onset RA	4
rheumatoid arthritis	1 patient	200 mg p.o.daily	No benefit, no adverse effect	5
rheumatoid arthritis and spondilarthopathies	12 patients	100-200 mg aqueous suspension or acetate pregnenolone i.m. daily	11 improved, no adverse effects	6
rheumatoid arthritis	16 patients	Double-blinded study. 500 mg pregnenolone divided in 5 doses daily p.o.	9 improved, but no advantage compared with placebo in 7 patients	7

rheumatoid arthritis	1 patient	300 mg pregnenolone i.m.	Improved, with remission after 3 weeks	8
rheumatoid arthritis	22 patients	350-400 mg pregnenolone i.m. daily	16 excellent improvement, 4 strong improvement , 2 poor response	9
rheumatoid arthritis	3 patients	50-100 mg pregnenolone i.m. daily	All improved (high disease activity scores at the start)	10
rheumatoid arthritis	40 patients	400-600 mg daily	30 improved (10 remission), 10 no effect	11
rheumatoid arthritis, mixed spondylitis and peripheral arthritis	19 patients	100-300 mg oily pregnenolone acetate i.m. daily, 15-20 days	2 improved (1 strong), 16 patients no change	12
rheumatoid arthritis	20 patients	300-1200 mg Δ-5 pregnenolone i.m. daily	15 improved (12 strong, 3 mild)	13
juvenile rheumatoid arthritis	1 patient	200-400 mg pregnenolone i.m. daily	Improved, with remission	14

References

1. Freeman, H., Pincus, G., Johnson, C.W., Bachrach, S., McCabe, G.E., and MacGilpin, H. Therapeutic efficacy of pregnenolone in rheumatoid arthritis. *J.A.M.A.*1950;142:1124-1128.
2. Pincus, G., and Hoagland, H. Effects on industrial production of the adminsitration of pregnenolone to factory workers. *J. Aviation Med.* 1944;15:98-115.
3. France, O., Losada, M., and Zanartu, J. Experience with pregnenolone in rheumatoid arthritis. *Rev Med Chil* 1951;79:572-574.
4. Limongelli, M.J., and Hadad, V.R. Pregnenolone in rheumatoid arthritis; study in 18 cases with this steroid. *Prensa Med Argent* 1953;40:3089-3092.
5. Schaposnik, F., and Gutierrez, A. Paradoxic effect of pregnenolone in rheumatoid arthritis. *Prensa Med Argent* 1951;38:1582-1584.
6. Davison, R., Koets, P., Snow, W.G., and Gabrielson, L. Effects of delta 5 pregnenolone in rheumatoid arthritis. *Archives of Internal Medicine* 1950;85:365-388.
7. Higgins, A.R., Jones, R.E., Jr., and Smith, T.W. The clinical effects of delta 5 pregnenolone in rheumatoid arthritis. *U S Armed Forces Med J* 1951;2:1717-1722.
8. Mattikow, B. 1951. Pregnenolone in arthritis. *N Y State J Med* 51:395.
9. Strazza, J.A. Treatment of rheumatoid arthritis with pregnenolone. *J Med Soc N J* 1950;47:472-475.
10. Bastenie, P.A., Franken, L., and Callebaut, C. Effects of pregnenolone on rheumatoid arthritis and rheumatic fever. *Brux Med* 1950;30:945-954.
11. McGavack, G.T. ACTH, cortisone and pregnenolone in arthritis and allied diseases. *Geriatrics* 1952;7:99-108.

12. Guest, C.M., Kammerer, W.H., Cecil, R.L., and Berson, S.A. Epinephrine, pregnenolone and testosterone in the treatment of rheumatoid arthritis. *J Am Med Assoc* 1950;143:338-344.

13. Cohen, A., Goldman, J., Dubbs, A.W., and Mc, B.T. A preliminary report of twenty patients treated with delta 5 pregnenolone and remissions in rheumatoid arthritis following gold therapy. *J Lancet* 1950;70:264-265.

14. Gaucher, M., and Truet, J. Chronic polyarthritis in children treated by delta 5 pregnenolone. *Rev Rhum Mal Osteoartic* 1952;19:689-690.
